# Study of the chemical interaction between a high-viscosity glass ionomer cement and dentin

**DOI:** 10.1590/1678-7757-2017-0384

**Published:** 2018-07-06

**Authors:** Shelyn Akari YAMAKAMI, Adriana Lemos Mori UBALDINI, Francielle SATO, Antonio MEDINA NETO, Renata Correa PASCOTTO, Mauro Luciano BAESSO

**Affiliations:** 1 Universidade Estadual de Maringá, Departamento de Odontologia, Maringá, Paraná, Brasil.; 2 Universidade Estadual de Maringá, Departamento de Física, Maringá, Paraná, Brasil.

**Keywords:** Glass-ionomer cement, Adhesion, Molecular structure, Fourier Transform Infrared Spectroscopy, Raman Spectrum Analysis

## Abstract

**Objective:**

To investigate the chemical interactions between a high-viscosity glass ionomer cement (GIC) (KetacTM Molar Easymix, 3M ESPE, Seefeld, Bavaria, Germany) and human dentin. It was also analyzed the dynamics of GIC setting mechanism based on the time intervals required for the GIC and the GIC mixed with dentin to achieve stability.

**Material and Methods:**

Each constituent of GIC – powder (P) and liquid (L) – and powdered dentin (D), as well as the associations P+L, D+L, and P+L+D in the concentrations of 29%, 50%, 65%, 78%, 82%, and 92% of GIC were analyzed with Fourier transform infrared (FTIR) and Raman spectroscopy.

**Results:**

New optical absorption bands and/or Raman bands, which were not present in P, L, or D, were observed in the associations. The concentrations of 29% and 50% of GIC showed higher interaction, revealing that the amount of dentin influences the formation of new optical absorption or scattering bands. FTIR bands showed that the setting time to achieve bond stability was longer for the high-viscosity GIC (38±7 min) than for the sample with 29% of GIC (28±4 min).

**Conclusions:**

The analysis revealed the formation of new compounds or molecular rearrangements resulting from the chemical interactions between GIC and dentin. Moreover, this study provides an effective method to evaluate the dynamics of the setting mechanism of GICs.

## Introduction

The glass ionomer cement (GIC) can be defined as a water-based material resulting from an acid-base reaction between a fluoroaluminosilicate glass powder and an aqueous polyacid solution.^2^ ^,^ ^7^ ^,^ ^22^ GICs have important properties such as adhesiveness to tooth structures, biocompatibility, thermal expansion coefficients close to teeth, and fluoride release.^18^ Adhesiveness is one of the main characteristics by which this material is indicated. GIC adhesion to tooth structures is described in the literature as a chemical interaction demonstrated by an ion-exchange layer in the tooth-restoration interface.^27^ This layer is formed during the setting reaction of the cement, in which the carboxylic radicals in the polyacrylic acid chelate the calcium ions present on tooth surface, producing a layer formed by calcium salts and aluminum polyacrylate.^15^ This interaction between polyalkenoic acid and dental substrate was first identified by Smith^19^ (1968) and further elucidated by Wilson and Kent^24^ (1972), who demonstrated the formation of ionic bonds on the tooth-restoration interface.

The presence of an intermediate layer on the interface formed by elements from both the cement and the tooth structure was demonstrated by Fourier Transform Infrared Spectroscopy (FTIR)^4^, and the intertubular diffusion of calcium ions of cement into dentinal structure was also observed with Raman spectroscopy.^1^ ^,^ ^22^ Furthermore, X-ray studies showed ionic exchanges between carboxylate ions in the polyalkenoic acid and the calcium in the tooth.^17^ ^,^ ^28^ However, the dynamics of the process, which could yield the time intervals required to achieve GIC and setting stability of GIC-dentin, as well as the characterization of the functional groups that may reveal the chemical interactions of the constituents of GIC with the dentin, were not investigated.

Therefore, since FTIR and Raman spectroscopy are techniques that characterize the chemical structure of materials such as dental structures and cements, this research aimed to investigate the formation of chemical interactions between the dentin and a high-viscosity GIC. This study also analyzed the dynamics of the GIC setting mechanism.

## Material and methods

We performed *in vitro* tests using a high-viscosity GIC (Ketac^TM^ Molar Easymix, 3M ESPE, Seefeld, Bavaria, Germany). Powdered dentin was obtained from human third molars extracted for orthodontic reasons, in accordance with the informed consent form signed by patients. This study was approved by the local Permanent Research Ethics Committee (resolution no. 681287 – CAAE no. 27637214.9.0000.0104).

For the analysis, measurements of each constituent of GIC – powder (P) and liquid (L) – and powdered dentin (D) were performed. Furthermore, measurements of the combinations GIC powder + GIC liquid (P+L), powdered dentin + GIC liquid (D+L), and GIC powder + GIC liquid + powdered dentin (P+L+D) in different concentrations (29%, 50%, 65%, 78%, 82%, and 92% of GIC) were also performed.

### Sample preparation

#### Human dentin (D)

Teeth were sectioned in the mesial-distal direction with a water-cooled diamond disc (Diamond Wafering Blade, Diamond Series 15HC, Arbon Size ½ - 12.7 cm, 10 cm x 0.3 mm, Buehler^®^, Illinois, Chicago, USA), fixed to a cutting machine (IsoMet^®^ 1000 Precision Saw, Buehler^®^, Illinois, Chicago, USA), and stored in a desiccator for 24 hours. We obtained powdered dentin using a spherical bur n^o^ 4, driven at low speed, positioned in the central part of the tooth, 0.3 mm from the enamel and pulp chamber.

#### GIC powder + GIC liquid (P+L)

P+L samples were obtained according to the manufacturer’s specifications, i.e., powder to liquid ratio of 2.4:1.

#### GIC powder + GIC liquid + Dentin (P+L+D)

Constituents of P+L+D were measured in an analytical balance (GH-202, A&D, Higashi-lkebukuro, Toshima, Tokyo, Japan) with precision of 0.0001 g. The samples were prepared in different concentrations of GIC and dentin to have a large variation between them. The amount of liquid was established to enable the mixing of the three components. Table 1 shows the different concentrations of the constituents of P+L+D.

**Table 1:** Ratio of glass-ionomer cement (GIC) samples weight (g)

Dentin:GIC	Dentin:GIC	Dentin:GIC	Dentin:GIC	Dentin:GIC	Dentin:GIC
2.4:1	01:01	0.54:1	0.28:1	0.23:1	0.09:1
29% GIC	50% GIC	65% GIC	78% GIC	82% GIC	92% GIC

#### Dentin + GIC liquid (D+L)

D+L samples were obtained by mixing powdered dentin to GIC liquid at a ratio of 2.4:1. This ratio was used to evaluate the dentin-liquid reaction using the same amount of dentin used to obtain the P+L+D samples.

Powdered portion was first mixed and then gradually incorporated into the liquid portion until a homogeneous mixture was achieved.^16^ The mixture was stored at 37°C and 100% relative humidity for 1 hour to allow the setting. Subsequently, the samples were ground in a mortar with agate pestle to be prepared for FTIR and Raman spectroscopy.

## Analysis of the chemical interaction

### Fourier transform infrared (FTIR) spectroscopy

The characterization of the molecular interactions between GIC and dentin was obtained with a VERTEX 70v FTIR research spectrometer (Buehler^®^, Illinois, Chicago, USA). Each sample was diluted in potassium bromide (KBr) and compressed under 10 tons for 1 min, to produce pellets. We collected KBr-sample pellets spectra using an average of 128 scans, with a 4 cm^-1^ resolution in the spectral range between 400 cm^-1^ and 4000 cm^-1^.

FTIR spectroscopy provides the vibrational modes of the molecules, evaluated by the optical absorption bands of the sample, which are considered fingerprints of specific molecules, allowing to obtain precise information about chemical changes in the material, the latter being assessed based on the possible changes in absorption bands and/or appearance of new bands.^5^

### Raman spectroscopy

Powdered samples were analyzed with the FT-Raman spectrometer (VERTEX 70v - RAM II module - Bruker^®^, Illinois, Chicago, USA), with a 4 cm^-1^ resolution and nominal laser power of 200 mW. Two hundred scans were performed to create each spectrum in the mid-infrared region (4000–400 cm^-1^). This technique provides the Raman bands, which also allows to detect the molecular vibrations of the samples. Therefore, FTIR and Raman spectroscopy might be considered complementary techniques to evaluate the molecular structure of the compounds of the samples.

We performed FTIR and Raman spectral analysis using the area of relevant band obtained by Gaussian deconvolution. In this mathematical evaluation, a band might be separated in distinct contributions, and their sum would represent the experimental band data.

## GIC setting dynamics

To investigate the setting dynamics, i.e., the time intervals required for the chemical bonds to stabilize, the P+L and the P+L+D mixture were used at 29% and 82% GIC concentrations, respectively. FTIR spectra were collected 10 min after mixing the constituents, and again every 10 min for 110 min. Samples were kept in FTIR spectrometer, maintaining the vacuum condition during the whole analysis. The spectral analysis was performed in the region between 4000–2750 cm^-1^, in which the measured bands can be associated to C-H and OH stretching. The evaluation was performed in terms of the variation of the band intensities as a function of time for GIC concentrations of 29%, 82%, and 100%. The normalization of the spectra was made at a peak of 1409 cm^-1^. The values of the areas were normalized at time zero (t=0) to better display the curve as a function of time. The area of the bands as a function of time, y(t), was adjusted by an exponential decay-type function conveniently written as:


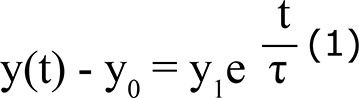


with y_0_ being the area of the bands after the material reached stabilization, y_1_ the amplitude of the area variation, t the instant times of the measurements, and τ the characteristic decay time representing the instant t when the area of the bands decay to 1/e (approximately 37% of y_1_ value). Mathematically, at the instant time when t=5τ, the curve shape tends to a constant value with time, meaning that 99.95% of the decay variation has been reached. After this instant of time, the material can be considered stabilized in terms of the changes of the chemical bonds. Therefore, the value of τ provides quantitative information on the dynamics of the changes in the material, allowing inspection of the physicochemical changes all over the setting process of GIC. This analysis was performed to observe the dynamics of possible changes in the chemical bonds of the restorative material and dentin, as well as their combinations.

## Statistical analysis

Statistical analysis was not carried out in this study due to the qualitative characteristic of the data resulting from FTIR and Raman spectroscopy, since these tests are considered fingerprints of specific molecules.

## Results

**Figure 1:**
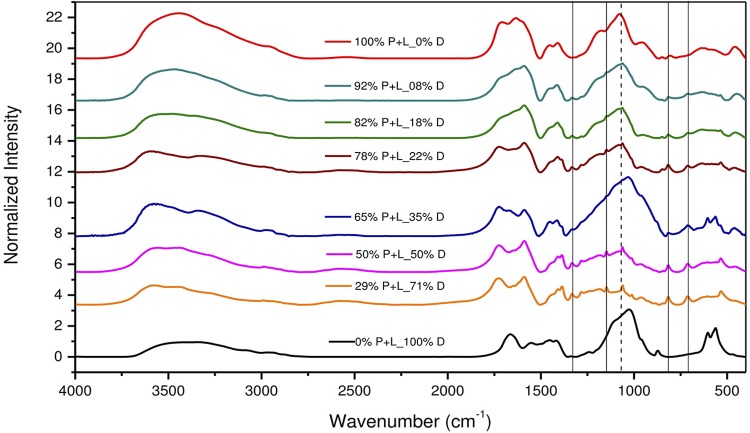
Fourier Transform Infrared Spectroscopy (FTIR) spectra of the different proportions of glass-ionomer cement (GIC) and dentin. The dashed visual guide shows the band of phosphate group at 1060 cm-1 and the lines of the emerged narrow bands

The results present the changes in the spectra that were more evident for concentrations of 29% and 50% of GIC, as shown by the orange and pink curves from data of the FTIR (Figure 1). The phosphate associated with the band around 1060 cm^-1^ almost disappeared in the samples with higher concentrations of dentin. The samples with 78%, 82%, and 92% of GIC showed a new band at 1060 cm^-1^ that increases proportionally to the GIC quantity until it resembles the GIC band displayed by the red curve.

**Figure 2:**
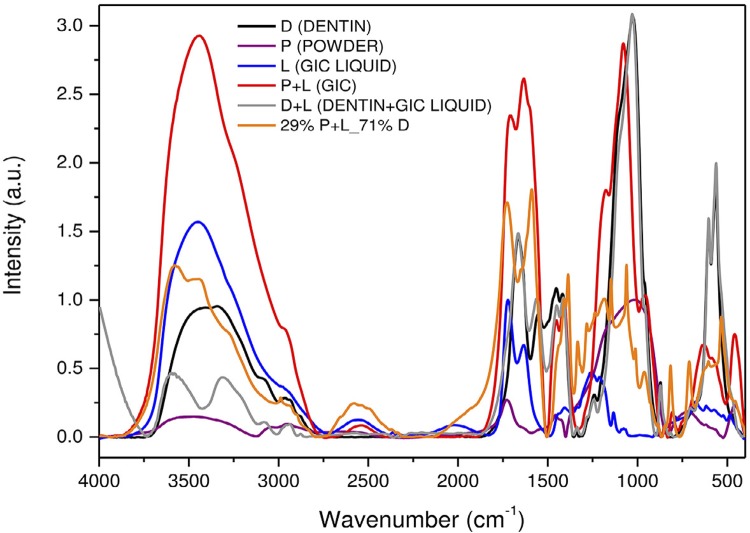
Fourier Transform Infrared Spectroscopy (FTIR) spectra of the precursors: glass-ionomer cement’s (GIC) powder (P), GIC’s liquid (L) and dentin (D). GIC’s combinations (P+L), dentin with the GIC’s liquid (D+L) and the sample of 29% GIC_71% Dentin (P+L+D)

**Figure 3:**
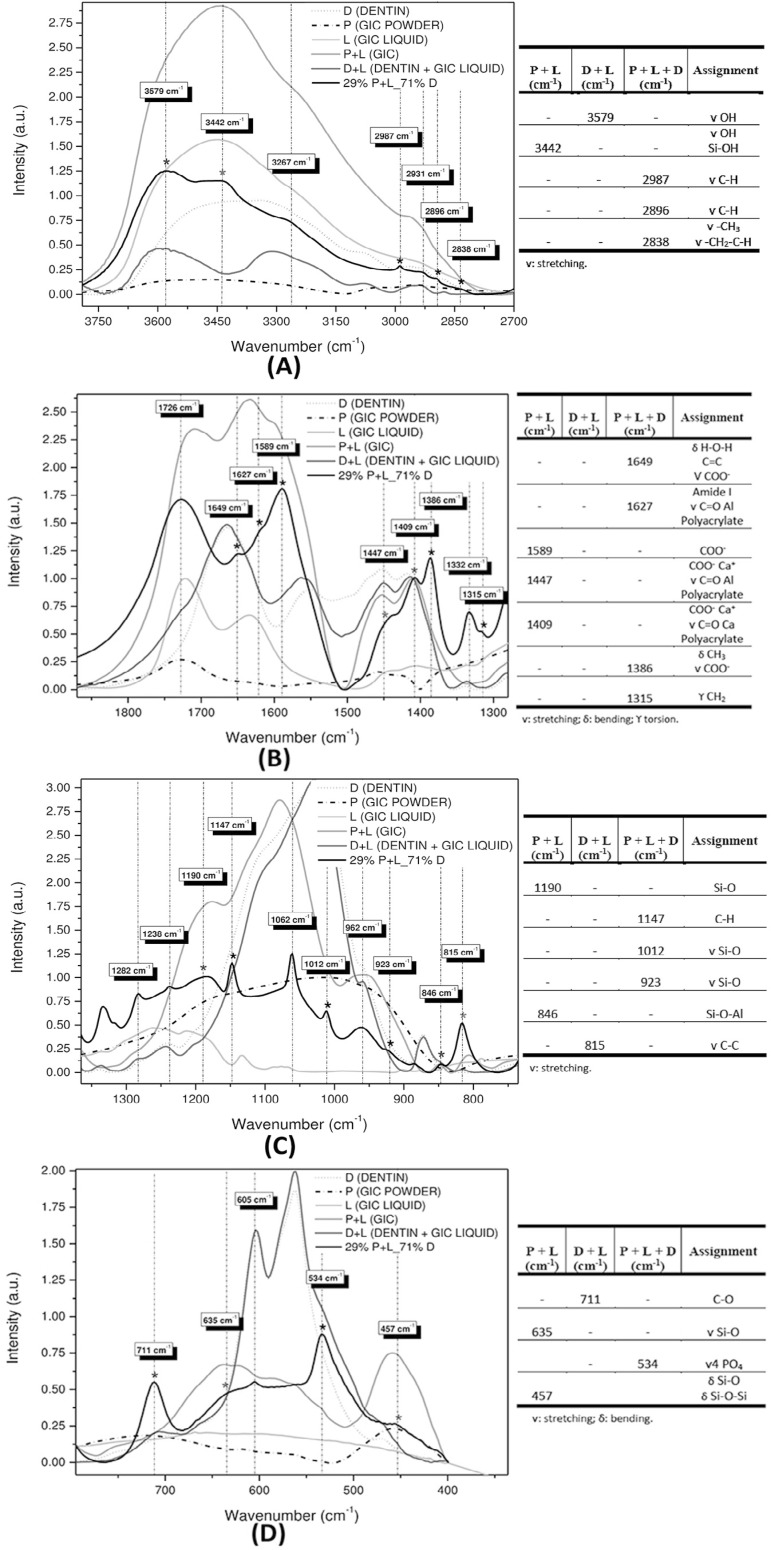
A: FTIR spectra in the region between 4000 and 2700 cm-1; B: Region between 1875 and 1300 cm-1; C. Region between 1300 and 750 cm-1; D: Region between 750 and 350 cm-1. The tables show the FTIR bands of the identified functional groups

**Figure 4:**
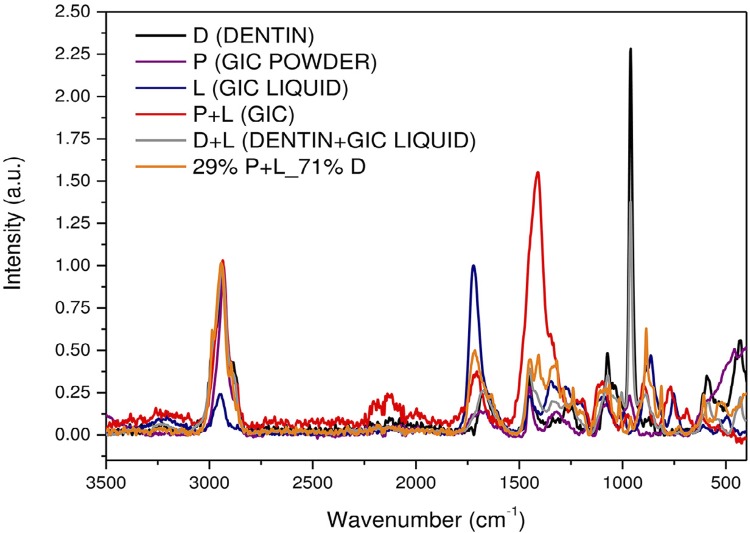
Raman spectra of the precursors: glass-ionomer cement’s (GIC) powder (P), GIC’s liquid (L) and dentin (D). GIC’s combinations (P+L), dentin with the GIC’s liquid (D+L) and the sample of 29% P+L_71% D (P+L+D)

**Figure 5:**
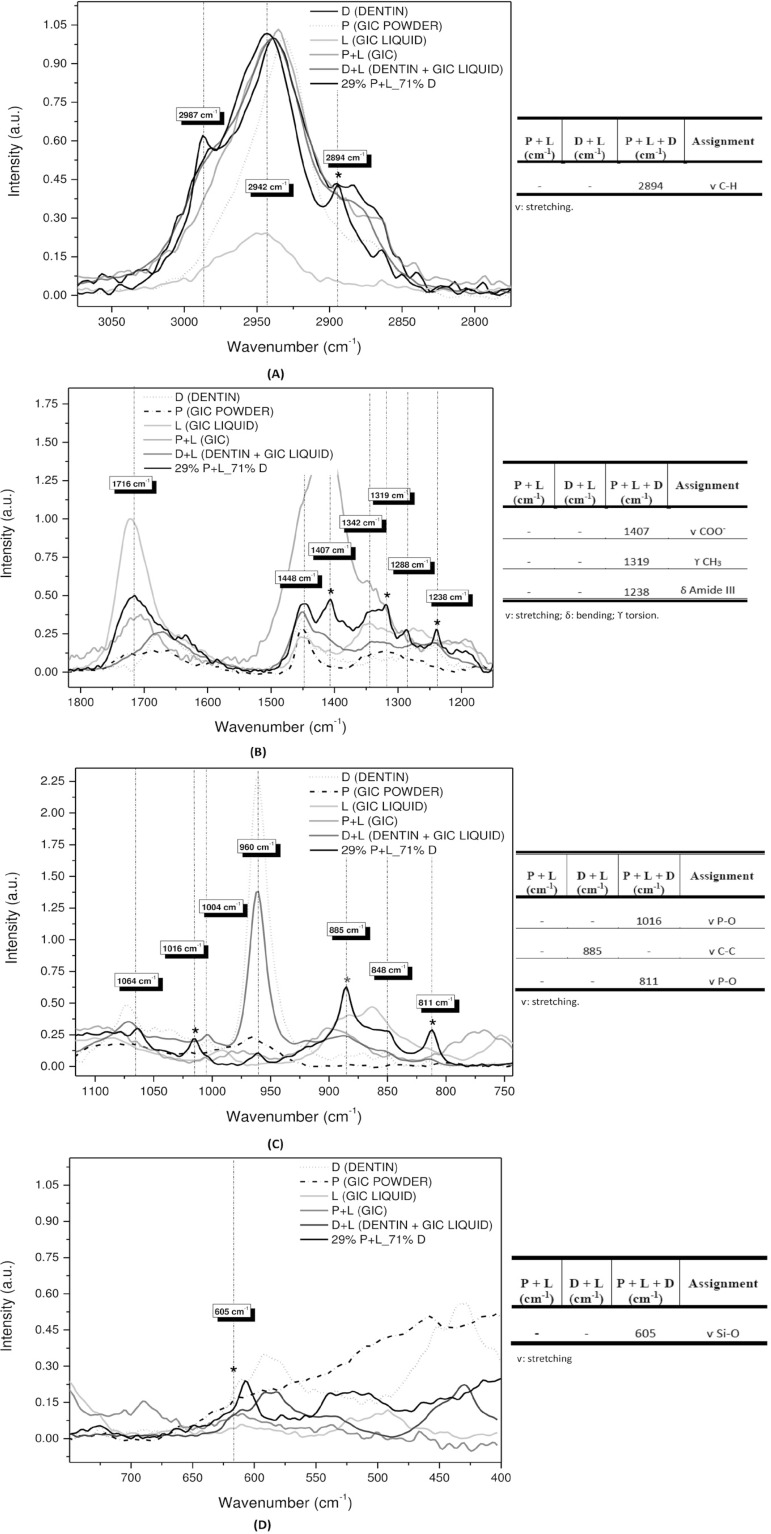
A: Raman spectra in the region between 4000 and 2700 cm-1; B: Region between 1875 and 1150 cm-1; C. Region between 1150 and 750 cm-1; D: Region between 750 and 350 cm-1; The tables show the Raman bands of the identified functional groups

Figures 2 and 4 show FTIR and Raman spectra of the precursors: GIC powder (P), GIC liquid (L), and dentin (D). Combinations of GIC (P+L), dentin with GIC liquid (D+L), and the chosen sample of higher dentin concentration, 29% P+L_71% D (P+L+D). For better visualization, these spectra were subdivided into four regions (Figures 3a to 3d and 5a to 5d). Next to each figure there is a table with the wavenumbers of each band and their associated functional groups. We can note the presence of FTIR and Raman bands formed by the P+L and L+D interactions and new bands (P+L+D) that were neither from dentin nor GIC.

Data from the FTIR of P+L mixture showed absorption bands at 3442, 1589, 1447, 1409, 1190, 846, 635, and 457 cm^-1^. The neutralization reaction (acid/base) appeared at 846 (Si-O-Al), 1190 and 635 (Si-O), and 457 (Si-O-Si) cm^-1^, with the silica gel (Si-OH) at 3442 cm^-1^. Carboxyl groups (COO^-^) were observed at 1589 and 1447 cm^-1^. The polyacrylate aluminum and calcium salts were characterized by the absorption bands at 1447 and 1409 cm^-1^, respectively.

The D+L interaction can be observed in the absorption bands at 3579, 815, and 711 cm^-1^ in the FTIR spectra, and in the Raman scattering band at 885 cm^-1^. The carboxyl groups of the polyalkenoic acid can be seen in the FTIR band at 711 cm^-1^ (C=O).

**Figure 6:**
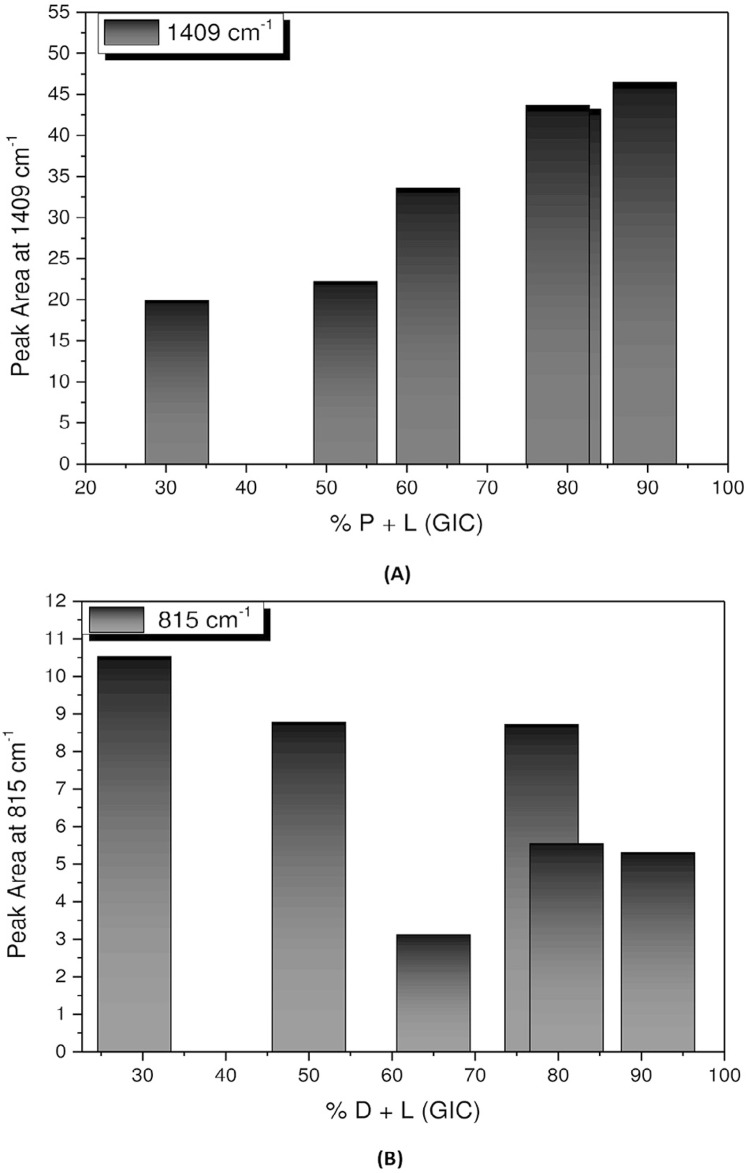
A: Variation of the band area at 1409 cm-1 as a function of glass-ionomer cement (GIC) (P+L) concentration. The arrow indicates the growing bonding behavior of the calcium polyacrylate salt; B: Variation of the band area at 815 cm-1 as a function of dentin + GIC’s liquid (D+L) concentration. The arrow indicates the decreasing bonding behavior of the organic compound

Figure 6(A) shows the predominant behavior of the P+L interaction from the FTIR band at 1409 cm^-1^, while Figure 6(B) shows the behavior of the D+L interaction from the FTIR absorption band at 816 cm^-1^.

**Figure 7:**
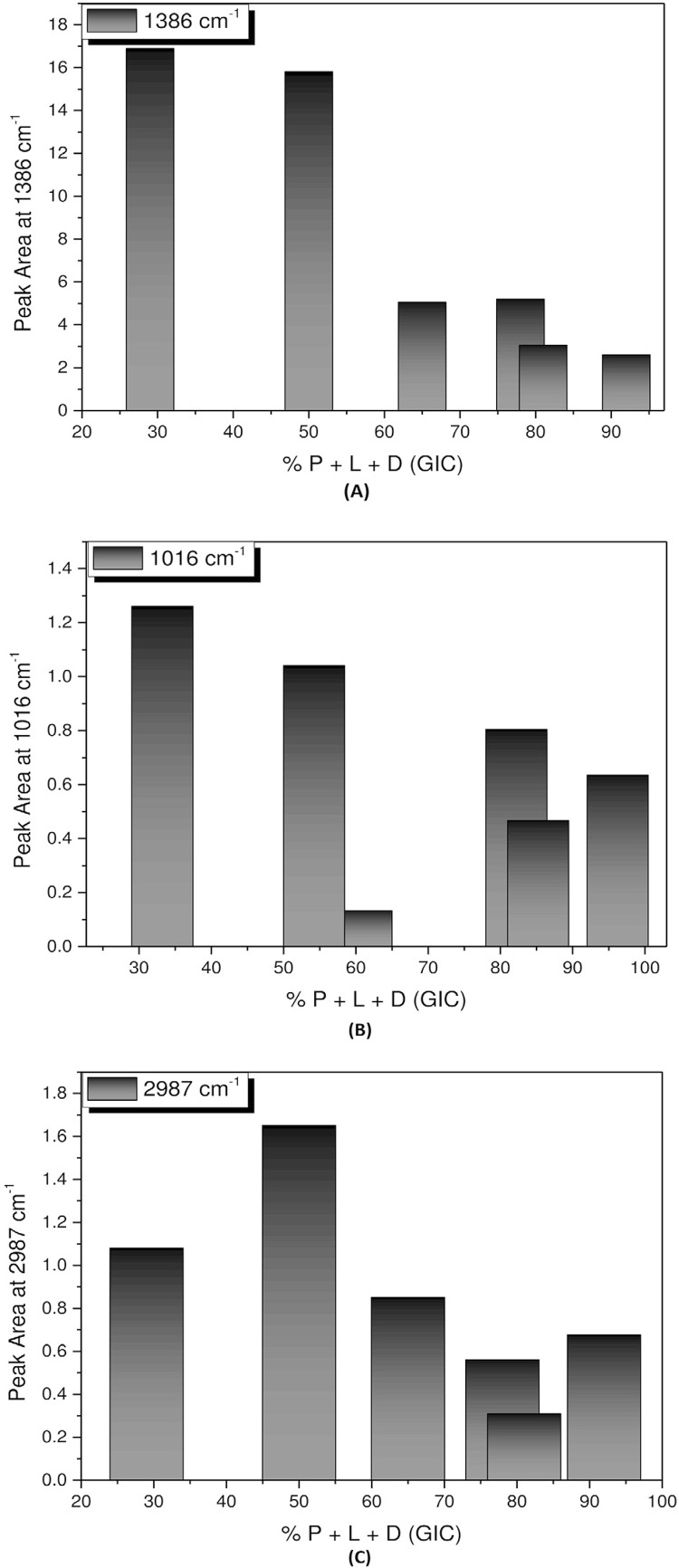
A: Variation of the band area at 1386 cm-1; B: Variation of the band area at 1016 cm-1; C: Variation of the band area at 2987 cm-1 as a function of GIC + dentin (P+L+D) concentration

Finally, the P+L+D interaction is evident in the bands at 2987, 2896, 2838, 1649, 1627, 1386, 1315, 1147, 1012, 923, and 534 cm^-1^ of the FTIR spectra, and at 2894, 1407, 1319, 1238, 1016, 811, and 605 cm^-1^ of the Raman spectra. Carboxyl groups can be observed in this study in the FTIR band at 1649 cm^-1^ and in the Raman band at 1407 cm^-1^ (COO^-^). On the other hand, the bands related to dentin are observed in the FTIR bands at 2987, 2896, and 2838 cm^-1^, and in the Raman band at 2894 cm^-1^ (C-H). The phosphate can be seen in the FTIR band at 534 cm^-1^, and in the Raman bands at 1016 and 811 cm^-1^ (P-O). The pendant metallic ions of the hydroxyapatite on the bridge between the carboxyl groups of the polyacid and the collagen molecules were observed in this study in the FTIR band at 1386 cm^-1^, and in the Raman band at 1319 cm^-1^ (CH_3_ bond). Figure 7(A) shows the behavior of the FTIR band at 1386 cm^-1^, 7(B) of the Raman band at 1016 cm^-1^, and 7(C) of the FTIR band at 2987 cm^-1^.

**Figure 8:**
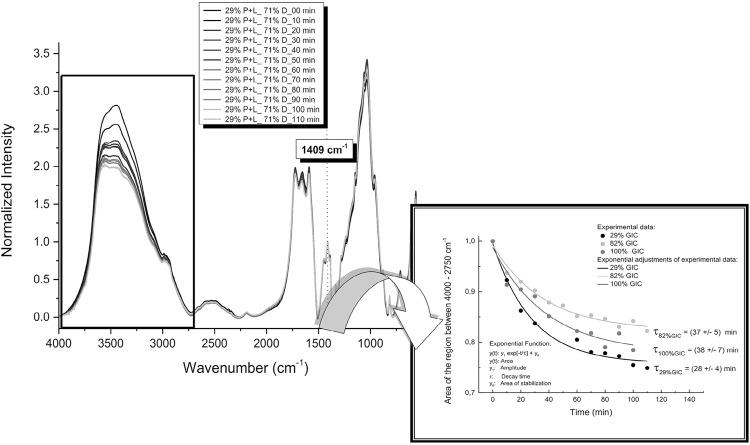
Fourier Transform Infrared Spectroscopy (FTIR) spectra of the sample 29% P+L_71% D as a function of time. The sample was measured up to 110 min, at intervals of 10 minutes. The spectra normalization of the spectra was at 1409 cm-1. The inset shows the area of the region between 4000-2750 cm-1 as a function of time for three concentrations of 100%, 82% and 29% of glass-ionomer cement (GIC). An exponential fitting with Eq (1) was performed to obtain the characteristic decay time (τ) of GIC interaction with dentin

Figure 8 shows the FTIR spectra as a function of time after the sample setting. The exponential fitting was performed with Eq (1) in the region presenting the highest variation of the areas of the bands. The results provided τ=38±7 min for the sample with 100% of GIC, 28±4 min for 29% of GIC and 71% of dentin, and 37±5 min for 82% of GIC and 18% of dentin.

## Discussion

This study showed the formation of new optical absorption bands, which revealed the occurrence of chemical interactions originated from the formation of new structures and/or molecular rearrangements of the high-viscosity GIC with dentin. These effects were dependent on the proportions of each material, as showed by the results of the FTIR (Figure 1).

We can note that changes in the spectra of the original materials were more evident in the specimens with higher amount of dentin because the characteristic band for it, i.e., the phosphate associated with the band at around 1060 cm^-1^ (FTIR), almost disappeared in these samples (concentration of 29% and 50% of GIC). If it was just a physical mixture (two or more compounds that not interact with each other), the higher the amount of dentin, the higher the band associated with the phosphate would be, since this mineral is abundant in the inorganic dentin. However, a reduction of this band was observed at the same time that several narrow bands at various spectral positions emerged. Different from the behavior presented by the samples with 29% and 50% of GIC, the samples with 78%, 82%, and 92% of GIC in the FTIR spectra (Figure 1) showed a behavior similar to a physical mixture. This mixture is represented by a spectrum with intermediate characteristics of GIC and dentin spectra, however, without new peaks.

This proportional interaction of the GIC with the dentin can be observed in the behavior of the band associated with the GIC, which is superposed in the same region at 1060 cm^-1^ (FTIR). As the amount of GIC in the samples increases, this band tends to proportionally increase to resemble the GIC band displayed by the red curve, indicating that there was no displacement or formation of new peaks. Thus, our results suggest that the samples with 29% and 50% of GIC showed the formation of more chemical bonds between dentin and the high-viscosity GIC. After visualizing the bonds from samples with different GIC-dentin proportions, all observed interactions will be discussed, taking as an example the sample with 29% of GIC and 71% of dentin, as shown in Figures 2 and 3.

In the P+L mixture, the neutralization reaction (acid/base) responsible for the gelation process of glass ionomer cements involves the acid hydrolysis of the Si-O-Al bonds in the vitreous network, which can be observed in the FTIR bands found in this study at 846 cm^-1^ (Si-O-Al), 1190 and 635 cm^-1^ (Si-O), and 457 cm^-1^ (Si-O-Si), demonstrating the formation of SiO_4_ and AlO_4_ tetrahedral linked by a Si-O-Al-type bond in the glass network.

When the Al^3+^ replace the Si^4+^ ions in the vitreous network, there is an unbalanced charge which is compensated by positive charges from modifier cations, such as the Ca^2+^ ions present in the glass composition. The oxygen atoms that connect the SiO_4_ and AlO_4_ tetrahedral structures make the glass network susceptible to acid attack, as these links are preferentially attacked by acid during cement setting. Therefore, such sites are required in the glass network so that the material can be vulnerable to acid attack and allow the formation of glass ionomer cement.^3^ ^,^ ^10^ ^,^ ^11^ ^,^ ^13^ ^,^ ^14^

Hydrolysis of these bonds will release Ca^2+^ and Al^3+^ cations and form the orthosilicic acid that is being gelled until forming silica gel. The absorption bands at 3442 cm^-1^ (Si-OH) in the FTIR spectra confirm that silica gel was formed by acid degradation, namely, the Si-O-Al bonds of the glass network were broken by the polyacrylic acid with the consequent incorporation of a water molecule. These bonds suggest that the degree of gelation in the silicate network keeps growing during cement setting.^3^ ^,^ ^11^ ^,^ ^12^ ^,^ ^16^ Note that the humidity of the environment was controlled to avoid the variation of this band, since there is free OH molecules bands in this same region.

The cations released from the glass network are then chelated by the carboxyl groups, which can be observed by the formation of absorption bands at 1589 and 1447 cm^-1^ (COO^-^) in the polymeric structure of the organic acid. After the neutralization reaction, they are gradually replaced by the bands related to the polyacrylate salts formed by the complexation of cations released from the glass network together with polyacrylic acid. The polyacrylate aluminum and calcium salts are characterized by the absorption bands at 1447 and 1409 cm^-1^, respectively. The cement at the end of the reaction appears to be composed by several glass particles coated with a layer of silica gel that are present in a matrix composed by polyacrylate salts, responsible for setting of cement.^3^ ^,^ ^10^ ^,^ ^13^ ^,^ ^14^ ^,^ ^18^

To strengthen the hypothesis abovementioned, the results of this study which demonstrate the occurrence of this type of interaction (P+L), are presented as follow: the band at 1409 cm^-1^ is a COO^-^ Ca^+^ bond (calcium polyacrylate), typically resulting from the GIC gelation reaction. As seen in Figures 4 and 5(A), this bonding interaction tends to increase as GIC powder is added, as demonstrated by the samples with 78%, 82%, and 92% of GIC, which presented higher bond intensity. It is suggested, then, that this is a P+L interaction.

The shape of the band tends to resemble the spectrum of 100% of GIC, and its growing pattern shows that this interaction increases as the amount of GIC powder is increased. This result confirms that cations released from the glass network were chelated by carboxyl groups to form calcium polyacrylate salts, thus demonstrating the gelation reaction of GIC. This is important from a clinical point of view, since it shows that a chemical reaction occurred in fact, and chemical bonds were established.

The D+L interaction occurred through the formation of ionic bonds between the carboxyl groups of the polyalkenoic acid, demonstrated by the FTIR band at 711 cm^-1^ (C=O), with the metal ions of the hydroxyapatite on the tooth surface. According to Wilson^21^ (1974), metal ions could form salt bridges between carboxylic acid pendant groups of the GIC and the positively charged hydroxyapatite on the tooth surface. We noticed that effective adhesion can be obtained by wetting the COOH groups that are free from the polyalkenoic acid that tends to initially form hydrogen bonds. These weak bonds are gradually replaced by ionic bonds during GIC gelation.

Our results showed that the D+L interaction was more evident in the samples with 29%, 50%, and 65% of GIC, i.e., the greater the amount of dentin, the better the visualization of the bands. We can suggest that this interaction depends essentially on dentin; as GIC powder is added, bond interaction tends to decrease. Chemically, the GIC powder competes to interact with available dentin powder. Figure 5(B) illustrates this interaction, showing the decreasing character of the chemical bond dependent on the amount of dentin.

The shape of the band tends to resemble the D+L band that appears in this same location. The intensity of this bond is typical of dentin, i.e., of the organic compound. The area decreases with increased amounts of GIC powder, demonstrating that the lower the amount of dentin in the sample, the lower the intensity of this bond. The hypothesis of being a pure dentin bond was discarded because neither the powdered dentin nor the GIC presented any bands in this same region. Thus, one may suggest that this bond is only formed when the dentin and the GIC liquid are placed together.

Finally, the P+L+D interaction is a result of one of the main properties of GICs, that is, their adherence to tooth structures. Therefore, previous studies^20^ ^,^ ^21^ pointed out that the inclusion of GIC in the conditioned dentin cavity promotes an initial polar attraction with predominance of weak hydrogen bonds with the free carboxyl groups that appeared after GIC handling, which are responsible for the shiny appearance of the material.

The interaction between carboxylic acid and tooth structure produces the displacement of the phosphate groups by carboxyl groups of the polyacid. As a result, positive charges are lost, such as the positive ions of calcium, to maintain electrical neutrality. Specifically, the carboxyl groups can be observed in this study in the FTIR band at 1649 cm^-1^ and in the Raman band at 1407 cm^-1^ (COO^-^). On the other hand, the bands related to dentin are observed in the FTIR bands at 2987, 2896, and 2838 cm^-1^, and in the Raman band at 2894 cm^-1^ (C-H). The phosphate can be seen in the FTIR band at 534 cm^-1^, and in the Raman bands at 1016 and 811 cm^-1^ (P-O). The ionized groups compete for a place on the dentin surface and for the cations to form crosslinks during cement gelation.^26^

Its dissolution on the enamel-dentin surface results in the buffering of the polyacid with the consequent increase in the local pH, associated with mineral precipitation that is deposited at the interface between the tooth and the cement. Bonding becomes permanent as the cement adheres to the tooth substrate through a multiplicity of adhesive groups that are connected by covalent bonds.^23^

Consensus exists about the fact that bonding mechanism to enamel is practically a process resulting from ionic or polar forces. However, when the bonding mechanism to dentin is considered, these interactions become more complex.^25^ McLean and Wilson^8^ (1977) suggested that bonding to the organic component would occur via hydrogen bonds or pendant metallic ions on the bridge between the carboxyl groups of the polyacid and the collagen molecules, which were observed in this study in the FTIR band at 1386 cm^-1^ and in the Raman band at 1319 cm^-^ ^1^ (CH_3_ bond).

Interestingly, the results of this study revealed the great influence of the amount of dentin in the formation of new bands. In other words, for these interactions occur, a minimum amount of dentin is required to react with the GIC. The study showed that most of the bands were more intense in the samples with concentrations of 29% and 50% of GIC. This means that when GIC powder is added to the sample, the intensity of the interactions decreased as function of the decreased dentin. Therefore, it is suggested that the formation of these bonds, or for them to be at least visualized, a greater amount of available dentin is necessary, as shown in Figure 7(A).

This figure shows that most of the new bands that emerged after mixing the GIC with dentin had a strong relationship with the amount of organic compound. The band at 1386 cm^-^ ^1^ is related to the vibrational modes of the C-H group, an organic component of dentin, thus, the higher the concentration of dentin, the higher the intensity of this interaction. This band is considered P+L+D because no bands referring to the P+L, D or D+L are present in this region.

Some results obtained with Raman spectroscopy revealed an interesting behavior, in which most scattering bands demonstrated borderline dentin proportions. That is, by increasing the amount of GIC, these bands decreased to some extent, usually in the proportions of 65%-78% of GIC, and as more GIC was added, an inversion in the intensity of this band took place. This suggests that the appearance of new bands reaches a limit, as shown in Figure 7(B).

In this figure, the L+P+D interaction is evident for concentrations of 29% and 50% of GIC, i.e., the greater the amount of dentin, the greater their interaction. This behavior is consistent, since this band is associated with the P-O bond present in dentin. When the amount of dentin is reduced, this bond also tends to decrease until it reaches the minimum concentration of 65% to 78% of GIC, considered a limit concentration for this bond. If the amount of dentin is diminished even further, this bonding interaction tends to P+L, the reason why the more GIC is added, the greater the interaction.

Other results showed a different behavior, as new absorption bands showed a saturation point rather than having a limit concentration. This means that the P+L+D interaction increases up to a certain point, after which it begins to decrease. The saturation points appear more frequently in the concentrations of 50% of CIV. Thus, the proportions of GIC and dentin seem to be determinant in the emergence or development of these new bands.

The figure 7(B) shows that L+P+D interaction tends to increase for 29% and 50% GIC concentrations, with the interaction reaching its maximum point with 50% of GIC. From this saturation point, as the amount of GIC is increased, the bond interaction decreases. This band is associated with the C-H group present in dentin and is considered a new band because it does not appear in other interactions.

Considering that this is an *in vitro* study, there are limitations regarding what happens in clinical conditions, particularly because of the use of powdered dentin. As the powdered dentin provides a much larger contact surface than dentin in its natural form, the chemical interaction with the GIC is maximized, that is, it is possible to find changes suggestive of chemical bonding that can occur in clinical practice with greater confidence. Not to mention the fact that the techniques used are sufficiently sensitive to identify these changes.

Another important point refers to the time used before the material was evaluated, that is, 1 hour after being manipulated. Most studies^6,9^ analyzed the chemical bonds in the samples after 24 h, considering this to be the time required for the material to acquire stability. However, although GIC setting may progress more slowly, the greatest variations in the material occur immediately after the preparation. Therefore, to better understand the dynamics of GIC setting with dentin, experiments were performed as a function of time with concentrations samples of 29%, 82%, and 100% of GIC, measuring the spectra every 10 min totaling 110 min. Figure 8 shows the FTIR spectra as a function of the measuring time. The exponential fitting with Eq(1) was applied on the time intervals presenting the highest variation of the areas of the bands.

As the material sets, note that there is a downward trend in the intensity of this band during the first 20 minutes. From this time, the band begins to exhibit saturation behavior, thus demonstrating the occurrence of slow changes, and the beginning of chemical bonds stability.

These findings suggest that the GIC tends to set more rapidly when in contact with the dentin. This seems to be related to the increased presence of minerals available for the reaction, which consumes the carboxyl groups of the cement faster. Thus, in clinical practice, it is important to guide the patient not to chew on the GIC restoration for at least 150 minutes (approximately five times the value of τ, which represents approximately 99% of the variation of the measure, according to the behavior of an exponential function). During this time, the material is still vulnerable and in the process of forming chemical bonds to the dentin. Thus, dentists must pay special attention when removing the matrix and not conduct finishing and polishing procedures immediately after the restoration to avoid breaking the newly formed GIG-tooth chemical bonds.

Within the limitations of this study, only one HVGIC was used, and with materials of different compositions the results could be different. On the other hand, these results demonstrated that the FTIR and Raman spectroscopy were able to identify the bonds in the physical mixture of the GIC with powdered dentin, significantly contributing to the elucidation of the chemical bonding mechanism. As there are few studies that characterize the bonds in this process, further research is still required to elucidate this important mechanism for the dental practice. Thus, the results presented here may serve as reference for future research, as an effective method to evaluate the dynamics of the setting mechanism of GICs is presented.

## Conclusion

New bands were formed resulting from the glass ionomer cement and dentin interaction. It was also observed a close relationship between the emergence of new bands and the amount of dentin available, i.e., the higher the amount of dentin, the greater the likelihood of new bands being formed and, consequently, the greater the chemical interaction between the GIC and the dentin. Regarding the clinical practice, it was found that the setting time of high-viscosity GIC was longer than the GIC-dentin mixture. Furthermore, it was also observed that the setting time of high-viscosity GIC was longer than the GIC-dentin mixture, and the time for such bonds to achieve 99% stability was about 150 min. Based on the foregoing, this study suggests a methodology capable of interpreting data related to the study of the adhesion of various materials.
